# Synaptosomal-associated protein 25 mutation induces immaturity of the dentate granule cells of adult mice

**DOI:** 10.1186/1756-6606-6-12

**Published:** 2013-03-12

**Authors:** Koji Ohira, Katsunori Kobayashi, Keiko Toyama, Hironori K Nakamura, Hirotaka Shoji, Keizo Takao, Rika Takeuchi, Shun Yamaguchi, Masakazu Kataoka, Shintaro Otsuka, Masami Takahashi, Tsuyoshi Miyakawa

**Affiliations:** 1 Division of Systems Medical Science, Institute for Comprehensive Medical Science, Fujita Health University, Toyoake, 470-1192, Japan; 2 Japan Science and Technology Agency (JST), Core Research for Evolutional Science and Technology (CREST), Kawaguchi, 332-0012, Japan; 3 Department of Pharmacology, Graduate School of Medicine, Nippon Medical School, Tokyo, 113-8602, Japan; 4 Center for Genetic Analysis of Behavior, National Institute for Physiological Sciences, Okazaki, 444-8585, Japan; 5 Division of Morphological Neuroscience, Gifu University Graduate School of Medicine, Gifu, 501-1194, Japan; 6 JST, PRESTO, Kawaguchi, 332-0012, Japan; 7 Department of Environmental Science and Technology, Faculty of Engineering, Shinshu University, Nagano, 380-8553, Japan; 8 Department of Biochemistry, Kitasato University School of Medicine, Sagamihara, 228-8555, Japan

**Keywords:** Epileptic seizure, Granule cell, Hippocampus, Immature dentate gyrus, Psychiatric disorder, Working memory

## Abstract

**Background:**

Synaptosomal-associated protein, 25 kDa (SNAP-25) regulates the exocytosis of neurotransmitters. Growing evidence suggests that SNAP-25 is involved in neuropsychiatric disorders, such as schizophrenia, attention-deficit/hyperactivity disorder, and epilepsy. Recently, increases in anxiety-related behaviors and epilepsy have been observed in SNAP-25 knock-in (KI) mice, which have a single amino acid substitution of Ala for Ser187. However, the molecular and cellular mechanisms underlying the abnormalities in this mutant remain unknown.

**Results:**

In this study, we found that a significant number of dentate gyrus (DG) granule cells was histologically and electrophysiologically similar to immature DG neurons in the dentate gyrus of the adult mutants, a phenomenon termed the “immature DG” (iDG). SNAP-25 KI mice and other mice possessing the iDG phenotype, i.e., alpha-calcium/calmodulin-dependent protein kinase II heterozygous mice, Schnurri-2 knockout mice, and mice treated with the antidepressant fluoxetine, showed similar molecular expression patterns, with over 100 genes similarly altered. A working memory deficit was also identified in mutant mice during a spontaneous forced alternation task using a modified T-maze, a behavioral task known to be dependent on hippocampal function. Chronic treatments with the antiepileptic drug valproate abolished the iDG phenotype and the working memory deficit in mutants.

**Conclusions:**

These findings suggest that the substitution of Ala for Ser187 in SNAP-25 induces the iDG phenotype, which can also be caused by epilepsy, and led to a severe working memory deficit. In addition, the iDG phenotype in adulthood is likely an endophenotype for at least a part of some common psychiatric disorders.

## Background

Synaptosomal-associated protein, 25 kDa (SNAP-25) is a soluble *N*-ethylmaleimide-sensitive factor attachment protein receptor (SNARE) protein that plays a pivotal role in regulating synaptic vesicle exocytosis [1-4]. In humans, several studies have suggested that SNAP-25 is involved in the verbal and performance intelligence quotient [5-7], learning, and memory [8,9]. A family-based study found that 3 single-nucleotide polymorphisms (SNPs: rs363039, rs363050, and rs362602) in the SNAP-25 gene are associated with the intelligence quotient [5,6]. One of these SNPs, rs363039, is associated with working memory capacity [7]. Additional SNPs at *Dde*I (rs1051312) [8] and *Mnl*I (rs3746544) [9] can affect working, verbal, and visual memory as well as attention/executive functions. In experimental animal studies, *SNAP-25* mRNA levels increase after induction of long-term potentiation (LTP) in the granule cells of the dentate gyrus (DG) [10]. Inhibition of hippocampal SNAP-25 results in impairment of long-term contextual fear and spatial memories and a decrease in LTP [11]. Moreover, selective inhibition of SNAP-25 expression prevents axon elongation and the transformation of growth cones into synaptic terminals [12], especially in hippocampal neurons [13].

Recent human genetic studies have discovered associations between SNAP-25 and various psychiatric and neurological disorders, such as schizophrenia [14-17], attention-deficit/hyperactivity disorder (ADHD) [18-24], and epilepsy [25-29]. Moreover, translational convergent functional genomics has demonstrated that SNAP-25 is one of top 42 candidate genes for schizophrenia [30]. Using a dominant-negative SNAP-25 mutant (SNAP-25 knock-in [KI] mice), in which Ser187 is replaced with Ala, we have previously shown that this mutation results in an increase in anxiety-like behaviors and epileptic seizures [29]. However, it remains unknown how this specific SNAP-25 mutation affects the neuronal properties and functions of the brain. The current study addresses this question using histological, gene expression, and electrophysiological analyses in SNAP-25 KI mice.

## Results

### Immature state in DG cells in SNAP-25 KI mice

**Figure 1:**
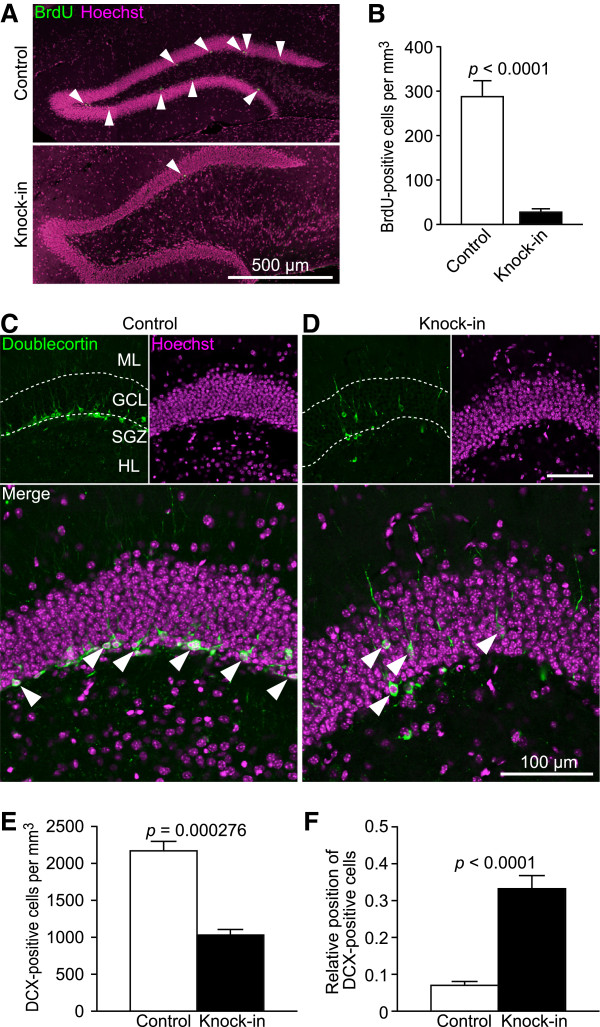
**Decrease in adult hippocampal neurogenesis in mutant mice.** (**A**, **B**) Cell proliferation was analyzed using BrdU staining (n = 6 mice each). Arrowheads indicate BrdU-positive cells. (**C**, **D**) Images of doublecortin (DCX)-positive cells (arrowheads) in the GCL are shown. ML, molecular layer. SGZ, subgranular zone. HL, hilus. (**E**) Quantification of DCX-positive cell numbers in the GCL (n = 4 mice each). (**F**) The positions of DCX-positive cells are shown as relative values between the subgranular layer (SGL; 0) and the border with the molecular layer (ML; 1) (n = 4 mice each).

Using Nissl staining and immunohistochemistry for NeuN, the cytoarchitecture of the brains of SNAP-25 KI mice was examined for abnormalities. In Nissl-stained sections, few differences were detected between mutant and control mice in terms of the cytoarchitecture of all brain regions examined, except in the hippocampus (Additional file 1: Figure S1A). Interestingly, the size of the hippocampal DG was significantly larger in mutants than in control mice (*p* < 0.0001; Additional file 1: Figure S1). The size of the hilus region was expanded in the DG of mutant mice. However, there was no obvious difference in the thicknesses of the granule cell layer (GCL) between controls and mutants. Although the mechanism that enlarges the DG region of mutants remains unclear, it is unlikely to involve alteration of adult neurogenesis, which was significantly decreased in mutant mice, as described in detail below (see Figure 1 and Additional file 1: Figure S3). However, it is possible that increased neurogenesis and/or gliogenesis during development and/or decreased cell death during development and adulthood may account for these effects. When stained with a neuron marker, NeuN, NeuN immunoreactivity in the DG of mutants was reduced to 60% of the control level (*p* < 0.0001; Additional file 1: Figure S1). The NeuN immunoreactivity seemed to be expressed at lower levels in individual granule cells in the DG of mutants (Additional file 1: Figure S1B, C). This decrease in NeuN immunoreactivity was not detected in other brain regions, such as the frontal cortex (*p* = 0.863), motor cortex (*p* = 0.742), somatosensory cortex (*p* = 0.928), striatum (*p* = 0.822), and thalamus (*p* = 0.673).

**Figure 2:**
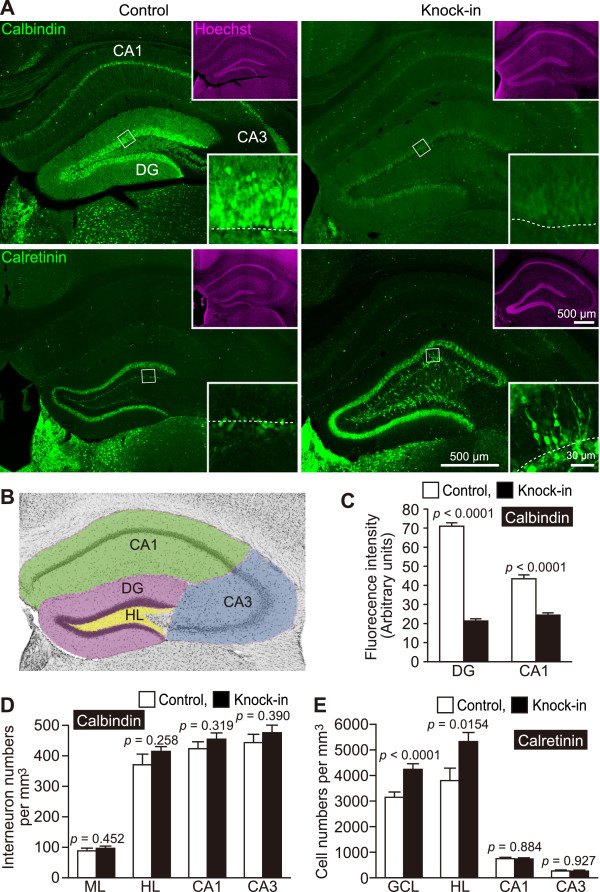
**Decreases in the expression levels of mature granule cell markers in mutant mice.** (**A**) Immunoreactivity for calbindin (green), a mature granule cell marker, and calretinin (green), an immature granule cell marker. The dotted line illustrates the border between the GCL and the hilus. (**B**) Definition of the hippocampal subregions measured in **C**–**E**. (**C**–**E**) Quantification of calbindin- and calretinin-positive cells in the hippocampal regions (n = 4 mice each) is shown.

Next, expressions of various cell markers were examined during neural cell development in the hippocampus, where adult neurogenesis occurs throughout life [31]. Neural stem cells exist near the subgranular zone, which is located between the hilus and the GCL. Neuroblasts generated in the subgranular zone are integrated into the deepest portion of the GCL, where they differentiate into granule cells and extend dendrites and axons [31]. During their development, new granule cells express various protein markers depending on the stage of cellular differentiation (Additional file 1: Figure S2). The fluorescence intensities of the mature granule cell marker calbindin were dramatically decreased in the DG and CA1 of mutants to 30% and 56% of control levels, respectively (Figure 2A, C). There were no significant differences between controls and mutants in the numbers of calbindin-positive cells in the DG molecular layer, hilus, CA1, and CA3 (*p* > 0.25). The number of cells positive for calretinin, an immature granule cell marker, in the GCL was significantly increased in mutants (Figure 2A, E). However, there were no significant differences between the mutants and controls in the numbers of calretinin-positive cells in the CA1 and CA3 regions, but there was a significant increase in the number of calretinin-positive cells in the hilus of mutants compared with that of controls (Figure 2E).

Because there was an increase in the number of immature granule cells in the GCL of mutants, adult hippocampal neurogenesis was evaluated using the cell proliferation markers Ki67 and bromodeoxyuridine (BrdU) and the late progenitor and early newborn granule cell marker doublecortin (DCX). In the DG of mutants injected with BrdU 3 days before perfusion, the numbers of BrdU- and Ki67-positive cells were significantly reduced (Figure 1A, B and Additional file 1: Figure S3). The number of DCX-positive cells was also significantly reduced in the mutants (Figure 1C–E). These findings indicate that adult hippocampal neurogenesis was decreased in the mutants.

The distribution of calretinin-positive cells within the GCL of mutants was also examined (Figure 2A). Calretinin-positive immature granule cells were primarily located in the subgranular zone (Figure 2A, bottom left). Because calretinin is also expressed in interneurons, which may be mislocated in mutant GCL, the distribution of immature granule cells in the DG was confirmed using DCX immunostaining [31]. As expected, DCX-positive immature granule cells were observed within the GCL (Figure 1C, D, F). As shown in Figure 2, the number of calretinin-positive immature granule cells was increased, and almost all of the granule cells in mutant mice were immunonegative for calbindin. These results suggest that the dentate granule cells remain in an immature state in adult SNAP-25 KI mice because of their failure to mature [32] or because of a reversal in maturation status (dematuration) [33].

### Transcriptome analysis of the DG of SNAP-25 KI mice

**Figure 3:**
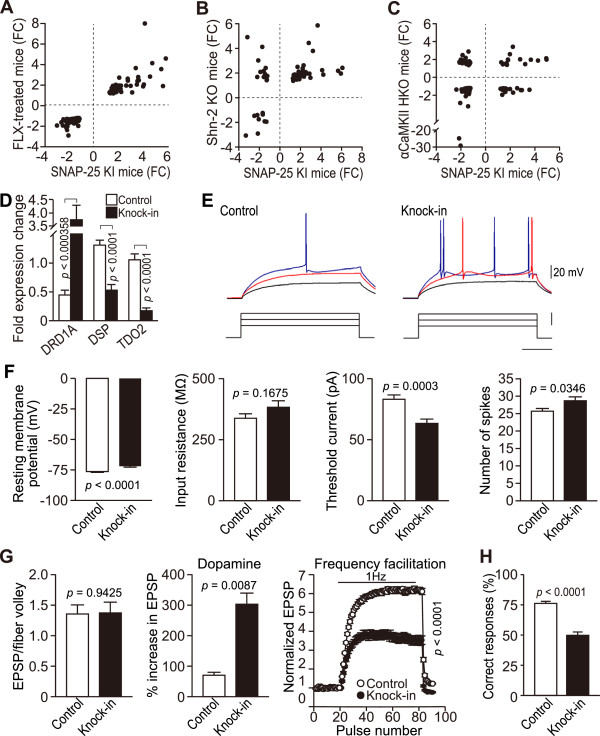
**iDG phenotype and severe working memory deficit in SNAP-25 KI mice.** (**A**–**C**) Scatter plot illustrating the fold change (FC) values for gene expression levels in the hippocampi of SNAP-25 KI mice (control: n = 7 mice, KI: n = 6 mice) and FLX-treated (**A**), Shn-2 KO (**B**), or αCaMKII HKO mice (**C**), which have the iDG phenotype. (**D**) mRNA expression analysis of *DRD1A*, *DSP*, and *TDO2*, which identify the iDG phenotype, using real-time PCR (control: n = 7 mice, KI: n = 9 mice). (**E, F**) The somatic physiological properties of granule cells in the DG of control (n = 22) and SNAP-25 KI mice (n = 21) are shown. (**E**) Sample recordings of granule cell action potentials evoked by depolarizing current pulses are shown. (**F**) Pooled data showing the resting membrane potential, input resistance, spike threshold current, and maximal number of spikes during sustained depolarization. (**G**) EPSP-to-fiber volley ratio (control: n = 9, KI: n = 8), synaptic potentiation induced by 10 μM dopamine (control: n = 5, KI: n = 4), and the frequency facilitation induced by a 1-Hz stimulation (control: n = 8, KI: n = 7) at the mossy fiber synapse are shown. (**H**) Severe working memory deficits were observed in SNAP-25 KI mice during a spontaneous alternation task in the T-maze (control: n = 9 mice, KI: n = 7 mice).

Previously, a method was established to reliably identify the immature DG (iDG) phenotype using a characteristic expression pattern of 3 genes [32-35], including the upregulation of dopamine receptor D1A (*DRD1A*) and the downregulation of desmoplakin (*DSP*) and tryptophan 2,3-dioxygenase (*TDO2*). Therefore, the mRNA expression patterns of *DRD1A*, *DSP*, and *TDO2* were examined in the hippocampi of mutant mice using quantitative real-time PCR analysis. A significant increase in *DRD1A* mRNA expression and significant decreases in *DSP* and *TDO2* mRNA expression were detected in mutants (Figure 3D), indicating that SNAP-25 KI mice have the iDG phenotype.

The iDG phenotype has been identified in alpha-isoform of calcium/calmodulin-dependent protein kinase II (αCaMKII) heterozygous knockout (HKO) mice [32,34-36], Schnurri-2 (Shn-2) KO mice [37], and chronic fluoxetine (FLX)-treated mice [33,38,39]. To elucidate the molecular mechanisms responsible for the iDG phenotype in SNAP-25 KI mice, DNA microarray analysis was performed using the hippocampi of mutant mice. From this analysis, significant up- or downregulation of 4587 genes was detected in the mutant (*p* < 0.05, fold-change (FC) < –1.2 or > 1.2). The transcriptome patterns in the hippocampi of SNAP-25 KI mice were then compared with those patterns in αCaMKII HKO mice, Shn-2 KO mice, and FLX-treated mice. Hippocampal gene expression data was obtained for αCaMKII HKO mice (data available publicly on ArrayExpress, http://www.ebi.ac.uk/arrayexpress/, accession number: E-MEXP-962) [32], for Shn-2 KO mice [37], and for FLX-treated mice (obtained from Gene Expression Omnibus, http://www.ncbi.nlm.nih.gov/geo/, accession number: GSE6476) [40]. The data for Shn-2 KO mice has been submitted for publication by Takao et al. [37] and will be available on the ArrayExpress database in the near future (accession number: GSE41307). Interestingly, SNAP-25 KI mice and other mice possessing the iDG phenotype, especially the FLX-treated mice, had similar gene expression patterns (Figure 3A–C), with more than 100 comparable changes in gene expression. The genes satisfying the threshold for statistical significance, i.e., *p* < 0.01 and |FC| > 1.2, are plotted in Figure 3A–C (see Additional file 2 for the list of genes plotted in Figure 3A–C). The strongest correlation was identified between the gene expression data of SNAP-25 KI mice and FLX-treated mice (*r* = 0.92, *p* < 0.001; SNAP-25 KI vs. Shn-2 KO mice, *r* = 0.45, *p* < 0.001; SNAP-25 KI vs. αCaMKII HKO mice, *r* = 0.24, *p* = 0.012). Moreover, the fold changes in gene expression were remarkably similar between SNAP-25 KI mice and FLX-treated mice. These results indicate that similar molecular events may occur in SNAP-25 KI and FLX-treated mice.

### Granule cells in SNAP-25 KI mice exhibited immature functional features

Given that the molecular expression data reflects a change in the state of granule cell maturation in SNAP-25 KI mice, it was assumed that these cells were also functionally altered. Therefore, the electrophysiological properties of mutant granule cells were also examined. When compared with mature cells, young granule cells are more easily excited by somatic current injection [41,42]. Whole-cell current clamp recordings from granule cells revealed that mutant granule cells had a depolarized resting membrane potential and an intact input resistance (Figure 3E, F). Mutant granule cells appeared to have a lower threshold current for firing and an increased number of spikes during sustained depolarization in response to the current injection. Thus, SNAP-25 KI granule cells were more excitable (Figure 3E, F).

The properties of the dentate-to-CA3 signal transmission mediated by mossy fibers (MFs), which are the axons of granule cells, were then examined. There was no significant change in the basal synaptic efficacy (Figure 3G). Consistent with the upregulation of *DRD1* mRNA, dopamine-induced synaptic potentiation was enhanced in mutant mice (Figure 3G). The prominent frequency facilitation, presynaptic short-term plasticity that is characteristic of mature MF synapses, was greatly decreased in mutants (Figure 3G). These changes are very similar to the changes observed in mice treated with FLX, which exhibit a “dematurated” DG [33,38]. Taken together, these results suggest that the granule cells in SNAP-25 KI mice have physiological properties that are characteristic of immature cells.

### Severe working memory deficit in SNAP-25 KI mice

Previous reports have shown that SNAP-25 KI mice display increases in anxiety-like behaviors [29] and that the *SNAP-25* gene is associated with various neuropsychiatric disorders, such as schizophrenia [14-17], ADHD [18-24], and epilepsy [25-29]. Individuals with each disorder exhibit characteristic behavioral features, including decreased prepulse inhibition (PPI) in schizophrenia, working memory deficit in schizophrenia and ADHD, and social deficits in schizophrenia. To further investigate the behavioral phenotypes related to these disorders, mutants were subjected to a comprehensive battery of behavioral tests (Additional file 3) [43]. Regarding the physical characteristics of the mutants, there were significant decreases in body weight (*p* < 0.0001), grip strength (*p* < 0.0001), and wire-hanging time (*p* < 0.0080), but no significant difference in body temperature (*p* = 0.833) or response latency in the hot plate test (*p* = 0.1594), in comparison with controls. In the rotarod test, although mutants failed to exhibit any significant differences in the latencies to fall both on the first day (Trials 1-3) and the second day (Trials 4-6), mutant mice had a tendency to have shorter latencies to fall on the second day (Additional file 1: Figure S4; Genotype effect: the first day, *p* = 0.464; the second day, *p* = 0.095). The enhanced anxiety-like behavior was confirmed in the mutants using the light–dark transition test (Additional file 1: Figure S5). In the open field test, vertical activity and stereotypic counts were significantly lower in SNAP-25 KI mice (Additional file 1: Figure S6). SNAP-25 KI mice tended to travel longer than the control mice in the early period of the test. After the initial 20-min period, SNAP-25 KI mice spent most of their time in the central part of the chamber and their activity level was significantly reduced as compared with controls. The mutants displayed abnormal social interactions when placed in a novel environment (Additional file 1: Figure S7). In the mutants, the total duration of active contact increased, and the mean duration per contact decreased. Furthermore, the number of total contacts appeared to be larger for the mutants.

PPI, the phenomenon in which a weak prestimulus suppresses the response to a startling stimulus, is often decreased in schizophrenic patients compared to healthy controls [44]. In the mutants, the PPI for the acoustic startle response was noticeably reduced, with an intensity of 120 dB for the startle stimulus, compared to that of controls (Additional file 1: Figure S8). Mutants were also subjected to the Porsolt forced swim test, which is used to assess depression-like behavior [45]. There were significant decreases in the immobility of the mutants on days 1 and 2, compared to those of controls (Additional file 1: Figure S9). There were also significant differences between the genotypes in terms of the distance traveled on days 1 and 2.

The mutants exhibited a severe working memory deficit during a spontaneous alternation task in a modified automated T-maze (Figure 3H). The mean percentage of correct responses for mutant mice remained at the chance level (~50%), whereas the mean percentage of correct responses for the control mice was greater than 75%. Both the mutants and controls scored at a similar level in a left-right discrimination task in the T-maze, but the mutants showed a significantly lower percentage of correct responses than the controls during the reversal learning sessions (*p* = 0.0121; Additional file 1: Figure S10).

### Decrease in the expression of destabilized Venus under the *arc* promoter in SNAP-25 KI mice

**Figure 4:**
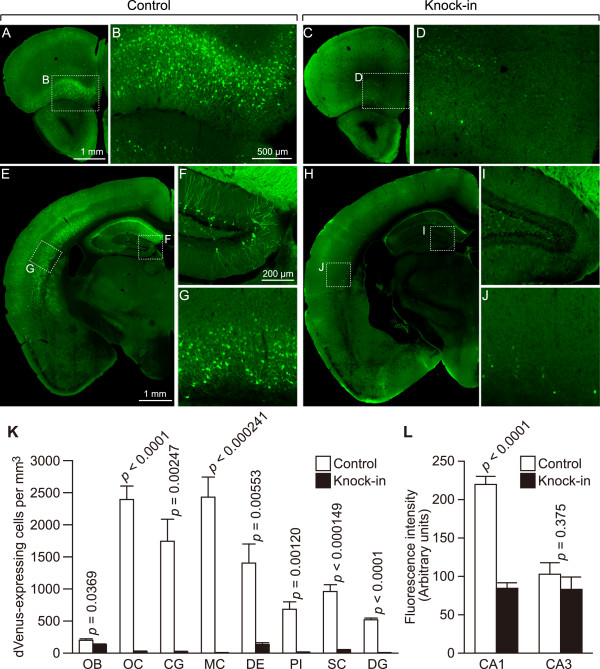
**Decrease in dVenus expression after exploration of a novel environment.** (**A**–**J**) Representative images of dVenus expression in control (**A, B, E–G**) and SNAP-25 KI mice (**C, D, H–J**) are shown. (**K, L**) dVenus expression levels in different regions of the brain (n = 3 mice each). OB, olfactory bulb; OC, orbitofrontal cortex; CG, cingulate cortex; MC, motor cortex; DE, dorsal endopiriform nucleus; PI, piriform cortex; SC, somatosensory cortex; DG, dentate gyrus; CA, cornu ammonis.

Taken together, our results indicate that the iDG phenotype is present in SNAP-25 KI mice. Therefore, it was expected that stimulation-induced activation of these cells would also be altered. To address this issue, Arc-dVenus transgenic mice were used that express the destabilized Venus (dVenus) fluorescent protein under the promoter of the immediate early gene (IEG) *arc* (a marker for mature in vivo activity-dependent responses in granule cells) [36,46]. Upon exposure to a new environment, SNAP-25 KI mice showed lower dVenus signals in almost all brain regions examined, especially in the DG and orbitofrontal cortex, compared with control mice (Figure 4).

### Rescue of the iDG phenotype and working memory deficit in SNAP-25 KI mice by valproate treatment

**Figure 5:**
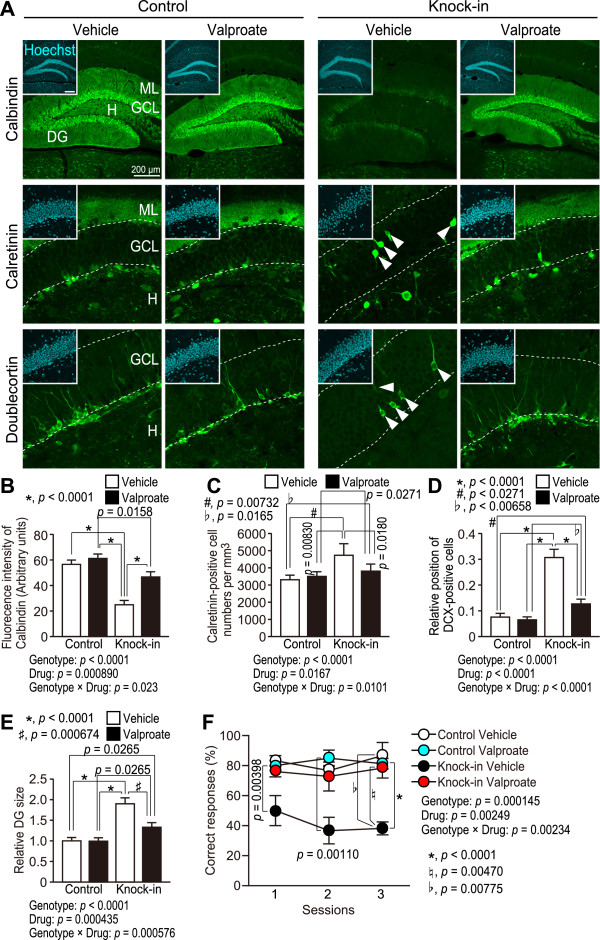
**Rescue of the immature state of the DG and working memory deficit in SNAP-25 KI mice by valproate treatments.** (**A**-**D**) Effects of chronic valproate treatment beginning on postnatal day 16 on the expression levels of calbindin, calretinin, and DCX in the DG (n = 3 mice each). (**E**) Effects of valproate administration on enlargement of the DG (n = 3 mice each). (**F**) Effects of valproate treatment on the working memory deficit in mutants (control vehicle: n = 3, control valproate: n = 8, KI vehicle: n = 3, KI valproate: n = 7).

We have previously reported that epileptic seizures occur in SNAP-25 KI mice [29]. Therefore, we investigated the ability of treatment with an antiepileptic drug, valproate, to rescue the iDG phenotype and working memory deficit in SNAP-25 KI mice. Mice were injected intraperitoneally with valproate (200 mg/kg body weight) every day for 4 weeks beginning at postnatal day 16. Interestingly, calbindin immunoreactivity was increased in valproate-treated SNAP-25 KI mice compared to that of vehicle-treated SNAP-25 KI mice (Figure 5A, *p* = 0.0158). However, the level of calbindin expression in the DG of valproate-treated SNAP-25 KI mice was not comparable to those of control mice (vehicle-treated control mice v.s. valproate-treated SNAP-25 KI mice: *p* = 0.0806; valproate-treated control mice v.s. valproate-treated control mice v.s. valproate-treated SNAP-25 KI mice: *p* = 0.0158). The number of calretinin-positive cells in valproate-treated SNAP-25 KI mice was decreased compared to that of vehicle-treated SNAP-25 KI mice (*p* = 0.0180), although there were significant differences in the number of calretinin-positive cells between valproate-treated SNAP-25 KI mice and vehicle- or valproate-treated control mice (Figure 5C, Genotype × drug: *p* = 0.0101; vehicle-treated control mice v.s. valproate-treated SNAP-25 KI mice: *p* = 0.0165; valproate-treated control mice v.s. valproate-treated control mice v.s. valproate-treated SNAP-25 KI mice: *p* = 0.0271). DCX-positive cells were located in the subgranular zone of valproate-treated SNAP-25 KI mice (Figure 5C, Genotype × drug: *p* < 0.0001; vehicle-treated control mice v.s. valproate-treated SNAP 25 KI mice: *p* = 0.0271; valproate-treated control mice v.s. valproate-treated control mice v.s. valproate-treated SNAP-25 KI mice: *p* = 0.00658). DGs from valproate-treated mutant mice were smaller than those in vehicle-treated mice (Figure 5E). Furthermore, a working memory deficit was not detected in valproate-treated SNAP-25 KI mice when compared with vehicle-treated or valproate-treated control mice (Figure 5F, Genotype × drug: *p* = 0.838).

## Discussion

This study demonstrated that the features of the DG neurons in SNAP-25 KI mice were strikingly similar to those of immature DG neurons in normal rodents. A severe impairment in the working memory of SNAP-25 KI mice was observed during the T-maze spontaneous alternation test, which is a behavioral task dependent on hippocampal function. Spontaneous, generalized seizures accompanied by epileptic discharges in both the cerebral cortex and hippocampus occurred after postnatal days 21–24 in SNAP-25 KI mice [29]. The results of this study also showed that valproate treatment rescued the iDG phenotype and working memory deficit in SNAP-25 KI mice. These results suggest that the Ser187 mutation in SNAP-25 causes the iDG phenotype and working memory deficit by inducing epilepsy. This interpretation is consistent with recent findings demonstrating that the pilocarpine-induced epilepsy model also causes the iDG phenotype [47]. However, the possibility that valproate rescues the phenotype in SNAP-25 KI mice in a manner independent of epilepsy cannot be excluded at this time.

We previously reported that there are no obvious differences in brain structures between control and SNAP-25 KI mice at 2.5 weeks of age [29]. In SNAP-25 KI mice, epilepsy is observed after postnatal day 21–25 [29], and, as observed in the current study, treatment with the antiepileptic drug valproate rescued the iDG phenotype, enlargement of the DG, and working memory deficit. Thus, these findings suggest that epilepsy after postnatal day 21–25 may convert the hippocampal granule cells to an immature state (i.e., dematuration) in SNAP-25 KI mice and enlarge the DG structure.

While the SNAP-25 mutant protein was constitutively expressed in mutant mice, it is not clear whether the iDG phenotype in SNAP-25 KI mice was caused by the cell autonomous role of SNAP-25. Ser187 of SNAP-25 is involved in a negative feedback mechanism for controlling neuronal excitability via inhibition of calcium dynamics [28]. Additionally, as already mentioned, SNAP-25 KI mice exhibit epileptic seizures after postnatal day 21–25. Thus, it is likely that the iDG phenotype is non-cell autonomously induced via epilepsy in SNAP-25 KI mice. However, we cannot exclude the possibility of cell autonomous roles for SNAP-25 in the iDG phenotype. Further studies will be needed to address cell autonomous and non-cell autonomous roles of SNAP-25 in the iDG phenotype.

The iDG phenotype has been reported in other mouse models (e.g., αCaMKII HKO [32], Shn-2 KO [37], and FLX-treated mice [33]), which exhibit hyperactivity and a severe working memory deficit. FLX-treated mice display increased anxiety-like behaviors [48]. In SNAP-25 KI mice, the iDG phenotype is characterized by common gene expression patterns (αCaMKII HKO mice have 204 altered genes in common; Shn-2 KO mice have 248 altered genes in common; and FLX-treated mice have 246 altered genes in common). Among the altered genes in SNAP-25 KI mice, 2 main patterns were identified: 1) upregulation of calretinin, brain-derived neurotrophic factor (BDNF), and glial fibrillary acidic protein (GFAP), and 2) downregulation of calbindin, TDO2, and DSP. Using patch-clamp analysis, granule cells in the iDG were also shown to exhibit electrophysiological features similar to those of immature granule cells, including a depolarized resting membrane potential and a lower threshold current for firing. An increased number of spikes during sustained depolarization was also observed, which is similar to the properties of dematurated granule cells after FLX treatment [33]. This result indicates that granule cells in the iDG phenotype are more excitable than mature granule cells. In contrast, differences in gene expression patterns and adult neurogenesis in the DG were discovered in mice possessing the iDG phenotype. In SNAP-25 KI mice, little adult neurogenesis can be seen, while adult neurogenesis is increased in αCaMKII HKO [32], Shn-2 KO [37], and FLX-treated mice [39]. Although the expression of *DRD1A* mRNA increased in all mouse strains and in FLX-treated mice, the magnitude of the increase in αCaMKII HKO and Shn-2 KO mice (< 2.5-fold) was smaller than in SNAP-25 KI and FLX-treated mice (> 7-fold). Therefore, these results suggest that future characterization of the iDG phenotype will include the above-described features along with properties that are currently unknown. Recently, increased expression of the immature granule cell marker calretinin was observed in the DG of patients with schizophrenia and bipolar disorder [49]. These data indicate that the iDG phenotype exists not only in mouse models, but also in humans with certain types of psychiatric disorders.

One of the behavioral abnormalities that mice with the iDG phenotype, such as SNAP-25 KI, αCaMKII HKO, and Shn-2 KO mice, have in common is a severe working memory deficit. This finding suggests that a working memory deficit is associated with the iDG phenotype. Using Arc-dVenus transgenic mice, we demonstrated in the current study that the expression of dVenus is dramatically decreased in all of the brain regions examined, especially in the DG and orbitofrontal cortex, which are involved in working memory. Anatomically, a monosynaptic pathway exists between the hippocampus and the medial prefrontal cortex. Axons from the hippocampal CA1/subiculum fields innervate the prelimbic/medial orbitofrontal cortex [50]. Disruption of the hippocampal-prefrontal pathway impairs the performance of rats in a spatial working memory task [51]. Furthermore, simultaneous tetrode recordings from the rat hippocampus and orbitofrontal cortex revealed that the activity in these different regions may be synchronized during a spatial working memory task [52]. Thus, the iDG phenotype may be the cause of the working memory deficit in the SNAP-25 KI mice in our study. A working memory deficit is known to be present in patients with schizophrenia [53], ADHD [54], and anxiety disorder [55]. The DG is reported to play an important role in working memory function [56]. Future studies, using different techniques such as conditional gene ablation and optogenetics, are needed to elucidate the exact causal relationship between the iDG phenotype and a working memory deficit.

The mechanism through which the iDG phenotype is induced is still unclear. However, previous studies have suggested that epilepsy is a candidate for producing the iDG phenotype. A long-term reduction in calbindin mRNA and protein expression has been observed in the hippocampi of rats following pilocarpine treatment [57]. A similar reduction in calbindin has been reported in human epileptic brains examined postmortem [58,59]. These findings suggest that epilepsy may induce the iDG phenotype by dematuration of mature granule cells. Furthermore, recent findings have shown that mice experiencing seizures induced by pilocarpine also exhibit the molecular characteristics of the iDG phenotype and associated behavioral abnormalities, which are similar to the abnormalities observed in αCaMKII HKO mice [47]. Moreover, valproate has been shown to suppress the iDG phenotype (present study, Figure 5) as well as seizures (previous study, ref. [60]). Taken together, this study suggests that the iDG phenotype in SNAP-25 KI mice may be induced by epilepsy and may result in behavioral deficits. However, the possibility that causes other than epilepsy may induce the iDG phenotype in SNAP-25 KI mice cannot be excluded. Future studies are needed to elucidate the exact relationship between the SNAP-25 mutation and the induction of the iDG phenotype.

The transcriptome data for SNAP-25 KI mice were similar to those of FLX-treated mice (see Figure 3A), suggesting that similar molecular events may occur in the DGs of SNAP-25 KI and FLX-treated mice. Therefore, one might wonder whether valproate treatment also reverses the FLX-induced dematuration phenotype. However, currently, it remains unclear whether valproate treatment can reverse the FLX-induced dematuration of hippocampal granule cells or enhance the effects of FLX on dematuration. It is of interest to examine whether valproate treatment enhances or reverses the effects of FLX on dematuration.

SNAP-25 KI mice may have endophenotypes other than the iDG phenotype in the brain. Endophenotypes which have been already found in mice with the iDG phenotype other than SNAP-25 KI mice, such as Shn-2 KO and FLX-treated mice, may be tested as a shortcut to identifying endophenotypes other than the iDG phenotype in the brains of SNAP-25 KI mice. Shn-2 KO mice show mild chronic inflammation in the frontal cortex and hippocampus, decreased expression of oligodendrocyte markers in the hippocampus, and reduced numbers of parvalbumin-positive neurons in the frontal cortex and hippocampal CA1 [37]. FLX-treated mice exhibit dematuration of the neurons in the visual cortex [61], amygdala [62], and DG [33], enhanced expression of BDNF [63], decreased adult neurogenesis in the subventricular zone [39,64], and induction of GABAergic interneurons from the neural progenitor L1-INP cells in the adult cortex [65]. It would be worthwhile to assess these endophenotypes in SNAP-25 KI mice in the future.

Psychiatric disorders, including anxiety disorder, ADHD, and schizophrenia [66-68], have been reported to accompany epilepsy. For example, 19% of patients with temporal lobe epilepsy are diagnosed with an anxiety disorder [69]. ADHD is another common psychiatric comorbidity in patients with epilepsy, and 20% of patients with epilepsy exhibit features of ADHD [70]. Furthermore, approximately half of all epileptic patients with psychosis are diagnosed with schizophrenia [71]. Interestingly, in a convergent functional genomics study identifying candidate genes for schizophrenia, 42 candidate genes were named [30]. Altered expression patterns were identified in SNAP-25 KI mice in 18 of these top 42 candidate genes for schizophrenia (Additional file 4) [30]. Strong evidence suggests that individuals with schizophrenia exhibit altered gene expression of *MIR137*, the gene encoding the miRNA miR-137 [72,73]. miR-137 expression is enriched in the hippocampus [74], especially in the DG [75]. A functional target of miR-137 is mind bomb-1 (MIB1), which facilitates neuron maturation [75]. In SNAP-25 KI mice, the expression of MIB1 was significantly decreased (Additional file 4), consistent with these previous studies.

Research investigating the relationship between the iDG phenotype and psychiatric disorders involving psychosis is in its infancy. However, recent studies have suggested that the iDG phenotype is involved in psychosis. The iDG phenotype has been observed in putative mouse models for schizophrenia, bipolar disorder, ADHD, and epilepsy [32,37,48,76]. A microarray analysis of post-mortem schizophrenic human brains revealed a significant reduction in hippocampal calbindin gene expression [77]. Previous reports have shown that the expression of calretinin, an immature granule cell marker, was significantly higher in postmortem brains of patients with schizophrenia and bipolar disorder compared to controls [49]. In addition, the increased expression of calretinin in patients with schizophrenia and bipolar disorder is closely associated with a diagnosis of psychosis and death due to suicide [49]. Therefore, it is likely that the iDG phenotype represents an endophenotype that causes cognitive deficits and psychosis, symptoms shared by these disorders. Tamminga et al. hypothesized that a reduction in glutamatergic transmission in the DG decreases the hippocampal pattern separation, which is a representation of inputs that have strong temporal and spatial similarities. This condition may result in false associations that govern some of the symptoms of psychosis, such as delusions and thought disorders [78]. The current results are consistent with this hypothesis.

In conclusion, our current data, together with previous findings, suggest that the iDG phenotype is responsible for some of the cognitive deficits observed in SNAP-25 KI, αCaMKII HKO, Shn-2 KO, and pilocarpine-treated mice and in patients with psychiatric disorders, such as schizophrenia, ADHD, and anxiety disorder.

## Methods

### Animals and drug administration

Mice heterozygous for the *snap-25*^*S187A*^ locus were bred to each other and maintained following standard husbandry procedures [29]. Genotyping of the mice was performed by PCR. Wild-type littermates were used as controls for the experiments.

Arc-dVenus transgenic mice have been described in previous studies [36,46]. SNAP-25 KI mice were bred onto a C57BL/6N background, and Arc-dVenus transgenic mice were maintained in the C57BL/6J background. SNAP-25 KI mice were crossed to Arc-dVenus mice, and the resulting F1 mice were intercrossed to generate F2 offspring (SNAP-25 KI/Arc-dVenus: SNAP-25 wild/Arc-dVenus).

BrdU labeling was performed following a previously described protocol [39]. Briefly, the animals were injected intraperitoneally with BrdU (Sigma-Aldrich, St. Louis, MO; 100 mg/kg body weight) every 24 h for 3 days prior to fixation.

The administration of valproate was initiated on postnatal day 16. Mice were given valproate (200 mg/kg body weight) by intraperitoneal injection at 10:00 a.m. each day for 4 weeks. Mice were fixed with 4% paraformaldehyde for 6 h after the last injection.

All of the animal experiments were approved by the Institutional Animal Care and Use Committee of Fujita Health University and Graduate School of Medicine, Kyoto University, based on the Law for the Humane Treatment and Management of Animals (2005) and the Standards Relating to the Care and Management of Laboratory Animals and Relief of Pain (2006). Every effort was made to minimize the number of animals used.

### Immunohistological analysis

Fixation and immunofluorescent staining were performed following a previously described protocol [79]. The sections were incubated at room temperature overnight with the indicated primary antibodies. After incubation with the secondary antibody, sections were mounted onto glass slides and embedded with Permafluor (Thermo, Waltham, MA). We used a confocal laser-scanning microscope (LSM 700; Carl Zeiss, Göttingen, Germany) to obtain images of the stained sections.

For NeuN immunohistochemistry, the sections were incubated at room temperature overnight in anti-NeuN antibody (diluted 1:1000, Millipore, Billerica, MA). Sections were then incubated with a biotinylated secondary antibody at room temperature for 2 h. The immunoreactive sites were visualized using the avidin-biotin complex peroxidase method with an avidin-biotin complex kit (ABC kit, Vector Laboratories, Burlingame, CA). A 3,3^′^-diaminobenzidine tetrahydrochloride, 4HC1 (DAB) solution containing 0.3% nickel ammonium sulfate in 0.05 M Tris–HCl, pH 7.6, was used as the substrate for peroxidase.

Quantification analysis was performed using a confocal microscope equipped with a 40× objective lens (Plan-NEOFLUAR, NA = 0.75, Carl Zeiss) and a pinhole setting that corresponded to a focal plane thickness of less than 1 μm. To avoid false positives caused by overlapping signals from different cells, randomly selected positive cells were analyzed by moving through the entire z-axis of each cell. Cells were counted under the live mode setting on the confocal microscope.

The number of immunoreactive cells was counted using ImageJ with the WCIF ImageJ bundle (http://www.uhnres.utoronto.ca/facilities/wcif/). Images from the confocal microscope were converted into 8-bit black-and-white images. Image thresholds were automatically determined by a plugin “maximum entropy threshold”, and binary images were generated. Once the images were segmented, the number of immunopositive cells was automatically generated using the command “Analyze/Analyze particles”. To exclude objects that were clearly not objects of interest in the binary image, the minimum size and maximum size were set to a range of 5–25 μm, which corresponds to the neuron cell body size.

### Antibodies

For primary antibodies, we used mouse monoclonal antibodies against calbindin (1:2000, Sigma-Aldrich), calretinin (1:10000, Millipore, Billerica, MA), NeuN (1:200 for immunofluorescent staining; 1:1000 for DAB staining), and parvalbumin (1:2,000, Sigma-Aldrich); a rat monoclonal antibody against BrdU (1:100, Abcam, Cambridge, MA); a rabbit polyclonal antibody against Ki-67 (1:10, Ylem, Avezzano, Italy), and a goat polyclonal antibody against DCX (1:200, Santa Cruz Biotechnology, Santa Cruz, CA). For secondary antibodies, we used anti-mouse IgG Alexa 488 (1:200, Invitrogen, Carlsbad, CA), anti-mouse IgG Alexa 594 (1:200, Invitrogen), anti-rat IgG Alexa 594 (1:200, Invitrogen), anti-rabbit IgG Alexa 488 (1:200, Invitrogen), anti-rabbit IgG Alexa 594 (1:200, Invitrogen), and anti-mouse IgG Biotin-conjugated (1:200, Vector Laboratories).

### Electrophysiology

Transverse hippocampal slices (20- to 32-week-old mice) were prepared, and electrophysiologic recordings were taken following a previously described protocol [32,33]. Mice were decapitated under halothane anesthesia, and both hippocampi were isolated. Transverse hippocampal slices (380 μm) were cut using a tissue slicer. Electrophysiological recordings were taken in a submersion-type chamber superfused at 2 mL/min with standard saline composed of the following (in mM): NaCl, 125; KCl, 2.5; NaH_2_PO_4_, 1.0; NaHCO_3_, 26.2; glucose, 11; CaCl_2_, 2.5; MgCl_2_, 1.3 (equilibrated with 95% O_2_/5% CO_2_) and maintained at 27–27.5°C. Whole-cell recordings were taken from granule cells in the DG using the blind whole-cell patch-clamp technique. Current-clamp recordings were taken using a pipette filled with a solution composed of the following (in mM): potassium gluconate, 140; HEPES, 20; NaCl, 8; MgATP, 2; Na_2_GTP, 0.3; EGTA, 0.05 (pH adjusted to 7.2 with KOH). The recording pipette was placed in the middle third of the GCL. Hyperpolarizing and depolarizing currents (400 ms) were injected through the recording pipette to measure the input resistance and to evaluate the action potential firing properties, respectively. Field excitatory postsynaptic potentials (EPSPs) at the MF-CA3 synapse were recorded in the stratum lucidum of the hippocampal CA3 region using a glass electrode filled with 2 M NaCl. Bipolar stimulating electrodes were placed in the GCL, and EPSPs were evoked at a frequency of 0.05 Hz unless otherwise specified. All recordings were taken using a Multiclamp 700B amplifier (Molecular Devices, Sunnyvale, CA), filtered at 2–10 kHz and stored in a personal computer using a digital interface (digitized at 5–20 kHz).

### Quantitative real-time PCR

Quantitative real-time PCR was performed following a previously described protocol [32]. Briefly, total RNA was isolated from the hippocampi of 3-month-old control and mutant mice. First-strand cDNA was prepared from 2 μg of DNase I-treated total RNA using SuperScript III reverse transcriptase (Invitrogen). PCR was performed using the DNA Engine Opticon 2 Real-Time PCR System (Bio-Rad, Hercules, CA) under the following conditions: 15 min at 95°C, followed by 45 cycles of 15 s at 94°C, 30 s at 60°C, 30 s at 72°C, and then 1 min at 65°C. β-Actin was amplified from all samples to normalize expression. The following primers were used: DRD1A (1–124), 5^′^-ATGGCTCCTAACACTTCTACCA and 5^′^-GGGTATTCCCTAAGAGAGTGGAC; TDO2 (1–105), 5^′^-ATGAGTGGGTGCCCGTTTG and 5^′^-GGCTCTGTTTACACCAGTTTGAG; DSP (7–113), 5^′^-GCTGAAGAACACTCTAGCCCA and 5^′^-ACTGCTGTTTCCTCTGAGACA; β*-*actin (851–962), 5^′^-AGTGTGACGTTGACATCCGTA and 5^′^-GCCAGAGCAGTAATCTCCTTCT.

### DNA microarray analysis

Microarray experiments were performed with hippocampi isolated from control and mutant male mice (25–40 weeks old, 7 control mice, 6 mutant mice) following a previously described protocol [32]. Briefly, RNA was isolated from brain tissues using the TRIzol method (Invitrogen), followed by purification using RNeasy columns (Qiagen, Valencia, CA). Double-stranded cDNA was synthesized from the total RNA, and an in vitro transcription reaction was then performed on biotin-labeled RNA that was generated using the cDNA. Labeled RNA was hybridized to the Mouse Genome 430 2.0 Array (Affymetrix, Santa Clara, CA), which contains 45101 probe sets, and the array was washed according to the manufacturer’s recommendations. The hybridized probe array was then stained with streptavidin-conjugated phycoerythrin, and each GeneChip was scanned using an Affymetrix GeneChip Scanner 3000 (GCS3000). The raw data were corrected for background using the robust multichip average (RMA) algorithm and quantile normalization [80] in the Affymetrix Expression Console 1.1 software. To determine whether there were differences in gene expression between 2 groups, a 2-tailed, unpaired Welch *t*-test was performed on the normalized data set. Only genes with *p*-values less than 0.05 and absolute values of FC greater than 1.2 were considered to be differentially expressed. Expression data and other information used in this paper will be available on the ArrayExpress database. Public microarray datasets were queried using NextBio [81], which is a database of microarray results (data used in Additional file 2 was accessed on February 1, 2012, and data used in Additional file 4 was accessed on May 25, 2012). NextBio is a repository of analyzed microarray datasets that allows the investigator to search results and expression profiles of publicly available microarray datasets. Gene overlaps were examined using Running Fisher tests.

### Exposure to a new environment

Before exposure to a new cage, each SNAP-25 KI/Arc-dVenus and SNAP-25 wild-type/Arc-dVenus mouse was housed in a standard cage for 24 h. For the new cage exposure, 3 mice were housed together in a new cage that included new paper tips, some types of nuts, and 2 neslets. Mice were euthanized and brain tissues were fixed 5 h after exposure to the new cages, when dVenus was expressed at its maximum level [36,46].

### T-maze spontaneous alternation task

The spontaneous alternation task was conducted using a modified T-maze apparatus and an automated video-tracking system (available through O’Hara & Co., Tokyo, Japan) [82,83]. The apparatus was constructed of white plastic runways with 25-cm high walls. It was partitioned into 6 areas by sliding doors that could be automatically opened by sliding downward. The stem of the T was designated as area S2 (13 × 24 cm), and the arms of the T were designated as areas A1 and A2 (11.5 × 20.5 cm). Areas P1 and P2 were the connecting passageways from the arms (area A1 or A2) to the start compartment (area S1). Mice were placed in the S1 area and immediately subjected to a forced-choice run (pseudo-randomly assigned to either left or right arms). Mice were held in either one of the arms (area A1 or A2) for 10 s. Thereafter, the door was opened so that the mouse could go back to the start area S1. They were held in the S1 area for 3 s and were then subjected to a free-choice run in which they were given access to both arms. This sequence (trial) was repeated 10 times per day (cutoff time, 7200 s). The intertrial intervals were 60 s. The percentage of trials in which mice entered the arm opposite to its forced-choice run during the free-choice run was calculated as the percentage of correct responses. Data acquisition and data analysis were performed using ImageTM software.

### Neuromuscular strength

Neuromuscular strength was tested using the grip strength and wire-hang tests. A grip strength meter (O’Hara & Co.) was used to assess the forelimb grip strength. Mice were lifted and held by their tails so that their forepaws could grasp a wire grid. The mice were then gently pulled backward by the tail, with their body parallel to the surface of the table, until they released the grid. The peak force applied by the mouse forelimbs was recorded in Newtons (N). Each mouse was tested 3 times, and the greatest value measured was used for statistical analysis. In the wire hang test, the mouse was placed on a wire mesh that was then inverted and gently waved to cause the mouse to grip the wire. The amount of time that passed before the mouse fell was recorded, with a 60-s cut-off time.

### Light/dark transition test

The light/dark transition test was conducted as previously described [84]. The apparatus used for the light/dark transition test consisted of a cage (21 × 42 × 25 cm) divided into 2 sections of equal size by a partition that contained a door (O’Hara & Co.). One chamber was brightly lit (390 lux), and the other chamber was dark (2 lux). Mice were placed in the dark side and allowed to move freely between the 2 chambers with the door open for 10 min. The total number of transitions between the chambers, the time spent in each side, the amount of time it took the mouse to first enter the light side, and the distance traveled were automatically recorded.

### Open-field test

Locomotor activity was measured using an open-field test. Each subject was allowed to move freely in the open-field apparatus (40 × 40 × 30 cm; AccuScan Instruments, Columbus, OH, USA) equipped with photocells (beam spacing 2.5 cm, beam diameter 4 mm, beam frequency 50 cycles/s). The total distance traveled, vertical activity (rearing measured by counting the number of photobeam interruptions), time spent in the center area of the open field, and counts of stereotypic behavior were recorded using the VersaMax system (AccuScan Instruments). The center area was defined as the central 20 × 20 cm portion. If a mouse stays in the area, then a mouse was considered to be in the center area. If a mouse broke the same beam (or set of beams) repeatedly, then it was considered to be exhibiting stereotypic activity. This type of activity is often exhibited as grooming or head bobbing behaviors. Stereotypic counts are the number of beam breaks that occur during any period of stereotypic activity. Data were collected for 120 min.

### Elevated plus-maze test

The elevated plus-maze test was conducted as previously described [85]. The elevated plus-maze consisted of 2 open arms and 2 enclosed arms of the same size (25 × 5 cm) with 15-cm high transparent walls. The arms and central square were made of white plastic plates that were elevated 55 cm above the floor. To minimize the likelihood of animals falling from the apparatus, 3-mm high Plexiglas walls surrounded the sides of the open arms. Arms of the same type were located opposite from each other. Each mouse was placed in the central square of the maze (5 × 5 cm) facing one of the closed arms. Mouse behavior was recorded during a 10-min test period. The number of entries into an arm and the times spent in the open and enclosed arms were recorded. The percentage of entries into open arms, time spent in open arms (s), number of total entries, and total distance traveled (cm) were analyzed. Data acquisition and analysis were automatically performed using ImageEP software.

### Hot plate test

The hot plate test was used to evaluate sensitivity to a painful stimulus. Mice were placed on a 55.0 ± 0.3°C hot plate (Columbus Instruments), and the amount of time that passed before the first hind-paw response was recorded. The hind-paw response was defined as either a foot shake or a paw lick.

### Social interaction test in a novel environment

The social interaction test was conducted as previously described [86]. Two mice of identical genotypes that were previously housed in different cages were placed into a box together (40 × 40 × 30 cm) and allowed to freely explore for 10 min. Their social behavior was monitored using a CCD camera. Analyses were automatically performed using ImageSI software. The total duration of contacts, the number of contacts, the number of active contacts, mean duration per of each contact, and total distance traveled were measured. The number of active contacts was defined as follows. Images were captured at a rate of 1 frame per second, and the distance traveled between 2 successive frames was calculated for each mouse. If the 2 mice contacted each other and the distance traveled by either mouse was longer than 5 cm, then the behavior was considered to be ‘active contact’.

### Rotarod test

Motor coordination and balance were tested using the rotarod test. The rotarod test, using an accelerating rotarod (UGO Basile, Comerio, Italy), was performed by placing mice on rotating drums (3 cm in diameter) and measuring the time each animal was able to maintain its balance on the rod. The speed of the rotarod accelerated from 4 to 40 rpm over a 5-min period.

### Startle response/prepulse inhibition tests

A startle reflex measurement system (O’Hara & Co.) was used to measure the startle response and prepulse inhibition. A test session began by placing a mouse in a plastic cylinder that was left undisturbed for 10 min. White noise (40 ms) was used as the startle stimulus for all trial types. The startle response was recorded for 140 ms (measuring the response every 1 ms) starting with the onset of the prepulse stimulus. The background noise level in each chamber was 70 dB. The peak startle amplitude recorded during the 140 ms sampling window was used as the dependent variable. A test session consisted of 6 trial types (i.e., 2 types for startle stimulus only trials and 4 types for prepulse inhibition trials). The intensity of the startle stimulus was 110 or 120 dB. The prepulse sound was given 100 ms before the startle stimulus, and its intensity was 74 or 78 dB. Four combinations of prepulse and startle stimuli were used (74/110, 78/110, 74/120, and 78/120 dB). Six blocks of the 6 trial types were presented in pseudorandom order such that each trial type was presented once within a block. The average intertrial interval was 15 s (range, 10–20 s).

### Porsolt forced swim test

The apparatus consisted of 4 Plexiglas cylinders (20 cm height × 10 cm diameter). The cylinders were filled with water (23°C) up to a height of 7.5 cm. Mice were placed in the cylinders, and their immobility and the distance traveled were recorded over a 10-min test period. Images were captured at a rate of 1 frame per second. For each pair of successive frames, the amount of area (pixels) that the mouse moved was measured. When the amount of area was below a certain threshold, the mouse behavior was judged to be “immobile.” When the amount of area equaled or exceeded the threshold, the mouse was considered to be “moving.” The optimal threshold used to judge mobility was determined by adjusting it to the amount of immobility measured by human observation. Immobility lasting less than 2 s was not included in the analysis. Data acquisition and analysis were automatically performed using the ImageJ-based original program, ImagePS (see “Image Analysis”).

### Y-maze test

Exploratory activity was measured using a Y-maze apparatus (arm length: 40 cm, arm bottom width: 3 cm, arm upper width: 10 cm, height of wall: 12 cm). Each mouse was placed in the center of the Y-maze field. The number of entries and changes in direction were recorded using ImageYM software. Data were collected for 10 min.

### Image analysis

The applications used for the behavioral studies (ImagePS, ImageLD, ImageEP, ImageTM, ImageYM, ImageSI) were based on the public domain programs NIH Image and ImageJ (developed at the U.S. National Institutes of Health and available on the Internet at http://rsb.info.nih.gov/ij/), which were modified for each test by Tsuyoshi Miyakawa (available through O’Hara & Co.).

### Statistical analysis

Statistical analysis was conducted using StatView (SAS Institute, Cary, NC, USA). Data were analyzed by one-way Analysis of Variance (ANOVA), two-way ANOVA, or two-way repeated measures ANOVA, unless otherwise noted. *Post hoc* analyses were performed on all ANOVAs found to be significant. The values in graphs are expressed as the means ± SEM. Effect sizes were calculated according to the Hedges’ *g*.

## Abbreviations

αCaMKII: Alpha-isoform of calcium/calmodulin-dependent protein kinase II; ADHD: Attention-deficit/hyperactivity disorder; DCX: Doublecortin; DG: Dentate gyrus; FLX: Fluoxetine; GCL: Granule cell layer; iDG: Immature dentate gyrus; KI: Knock-in; Shn-2: Schnurri-2; SNAP-25: Synaptosomal-associated protein, 25 kDa

## Competing interests

Tsuyoshi Miyakawa is an advisor/consultant for Astellas Pharma Inc. The other authors declare no conflicts of interests.

## Authors’ contributions

KO and TM conceived the study. TM led the project. KO performed the majority of experiments. KK performed the electrophysiological analyses. KT and HKN performed the transcriptome analyses. HS and KT performed the behavioral experiments. RT performed the immunohistological analyses and quantification. SY provided Arc-dVenus mice. MK, SO, and MT provided SNAP-25 KI mice. KO and TM co-wrote the paper. All authors read and approve the manuscript.

## Supplementary Material

Additional file 1: Figure S1 Enlargement of the hippocampal DG and decreased expression of NeuN in the DG of SNAP-­25 KI mice. **Figure S2.** Changes in the maturation of the granule cells generated in adults. **Figure S3.** Decrease in cell proliferation in SNAP-­25 KI mice. **Figure S4.** Physical characteristics of SNAP-­25 KI mice. **Figure S5**. Increased anxiety-like behavior in SNAP-25 KI mice during the light-­dark transition test. Supplementary **Figure S6.** Abnormal locomotor activity of SNAP-­25 KI mice in an open-­field test. **Figure S7.** Abnormal social behaviors in SNAP-­25 KI mice during a social interaction test. **Figure S8.** Normal startle response and decreased prepulse inhibition in SNAP-­25 KI mice. **Figure S9.** Decreased immobility time in SNAP-­25 KI mice in the Porsolt forced swim test. **Figure S10.** T-­maze left-­right discrimination task. Click here for file

Additional file 2 Genes differentially expressed in the hippocampi of SNAP-25 KI mice and either αCaMKII HKO mice, Shn-2 KO mice or FLX-treated mice. Click here for file

Additional file 3 Comprehensive behavioral test battery of SNAP-25 KI mice. Click here for file

Additional file 4 Common changes in gene expression levels in the hippocampi of SNAP-25 KI mice and FLX-treated mice and the dentate gyrus of Shn-2 KO mice and αCaMKII HKO mice. Click here for file
