# Supplementary material for: Synaptosomal-associated protein 25 mutation induces immaturity of the dentate granule cells of adult mice
Source: Mol Brain. 2013 Mar 12;6:12. doi: 10.1186/1756-6606-6-12 (PMC3605216; doi:10.1186/1756-6606-6-12)
Supplement: Additional file 1: Figure S1 — Enlargement of the hippocampal DG and decreased expression of NeuN in the DG of SNAP-­25 KI mice. Figure S2. Changes in the maturation of the granule cells generated in adults. Figure S3. Decrease in cell proliferation in SNAP-­25 KI mice. Figure S4. Physical characteristics of SNAP-­25 KI mice. Figure S5. Increased anxiety-like behavior in SNAP-25 KI mice during the light-­dark transition test. Supplementary Figure S6. Abnormal locomotor activity of SNAP-­25 KI mice in an open-­field test. Figure S7. Abnormal social behaviors in SNAP-­25 KI mice during a social interaction test. Figure S8. Normal startle response and decreased prepulse inhibition in SNAP-­25 KI mice. Figure S9. Decreased immobility time in SNAP-­25 KI mice in the Porsolt forced swim test. Figure S10. T-­maze left-­right discrimination task. [file 1756-6606-6-12-S1.pdf]

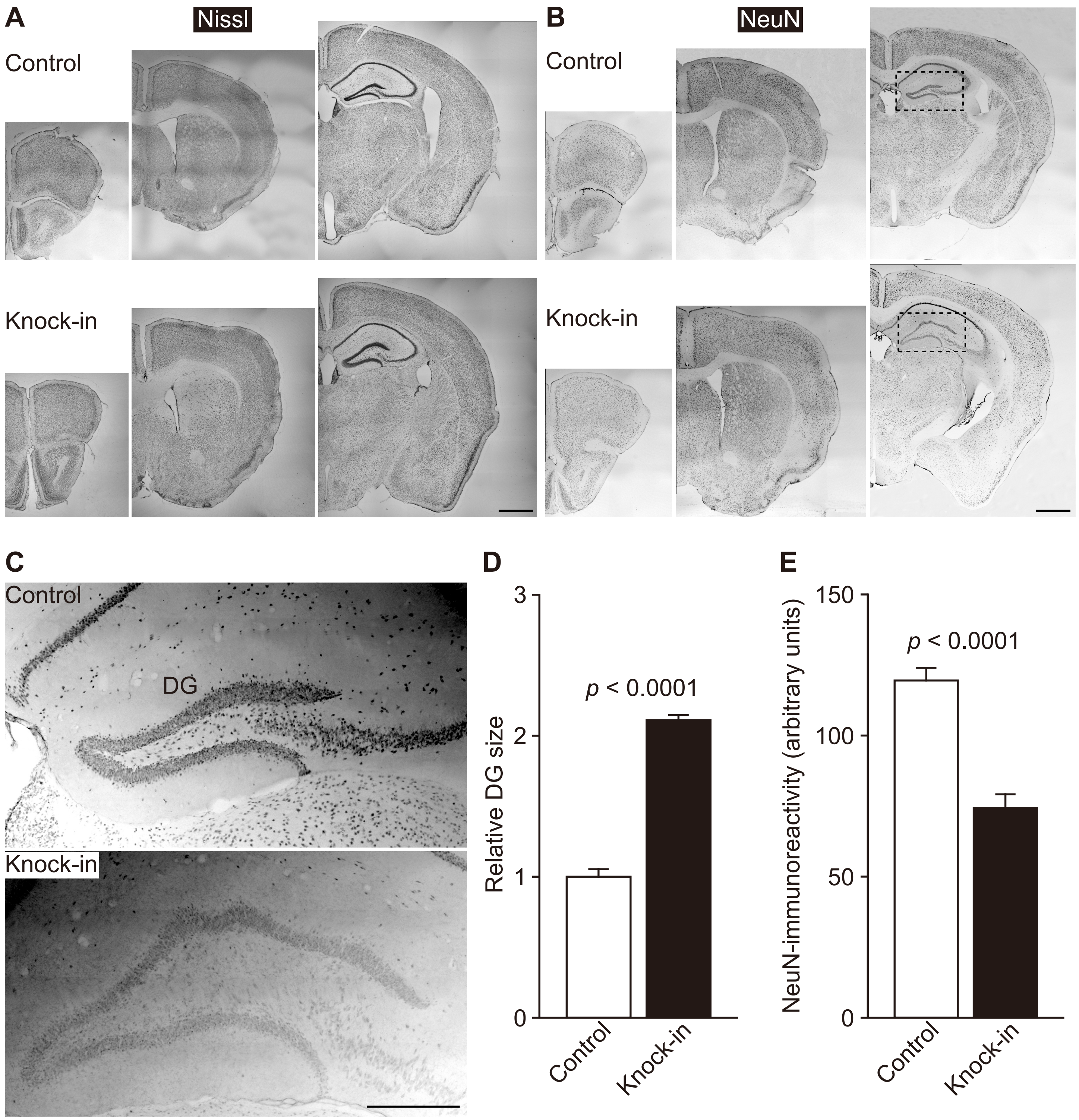

Supplementary Figure S1. Enlargement of the hippocampal DG and decreased expression of NeuN in the DG of SNAP-25 KI mice. (**A**) Histological analysis using Nissl staining shows an increase in the size of the DG in mutants. (**B**-**E**) The NeuN-immunoreactivity in the brain of controls (upper panels in **B** and **C**) and mutants (lower panels in **B** and **C**) are shown. Higher magnification images of the boxed-in areas in **B** are displayed in **C**. (**D**) The DG size of the mutants was larger than that of the controls. (**E**) The NeuN-immunoreactivity decreased by 60% in the DG of mutants compared with controls ($p < 0.0001$). Scale bars, 500 μm (**A**, **B**), 100 μm (**C**).

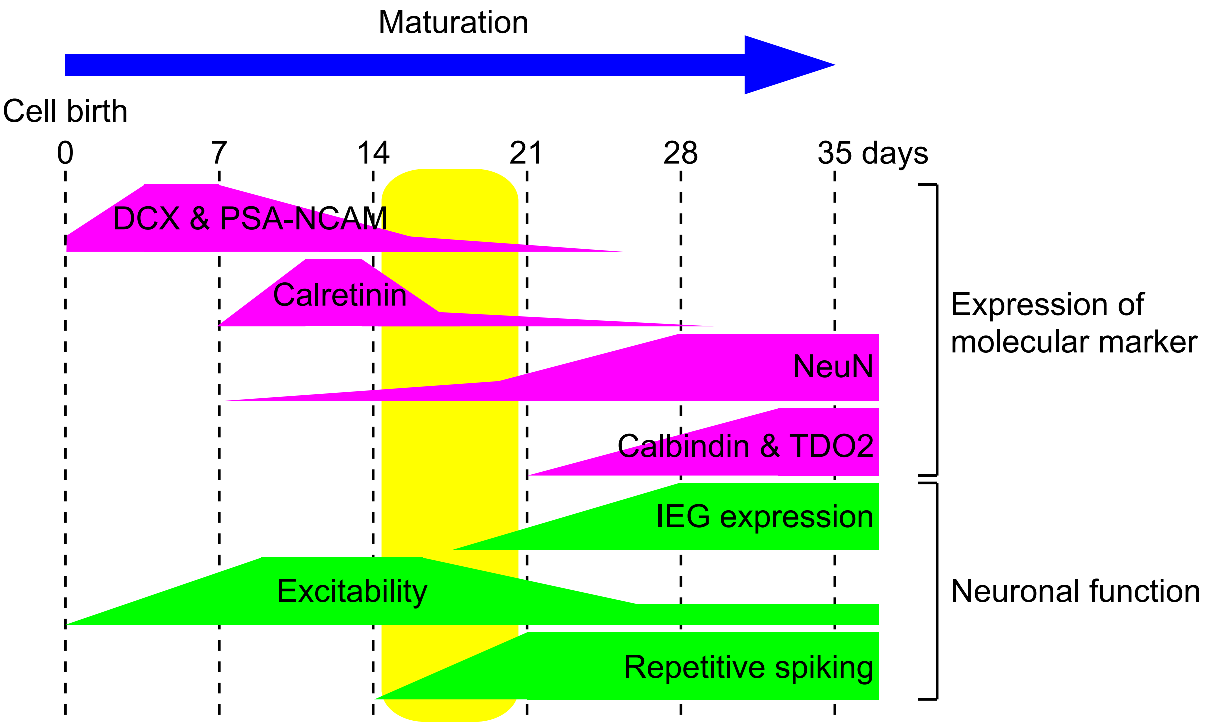

Supplementary Figure S2. Changes in the maturation of the granule cells generated in adults. Schematic representation of dentate granule cell maturation. The state of granule cells in SNAP-25 KI mice is comparable to that of 2- to 3-week-old adult-generated neurons (indicated in yellow).

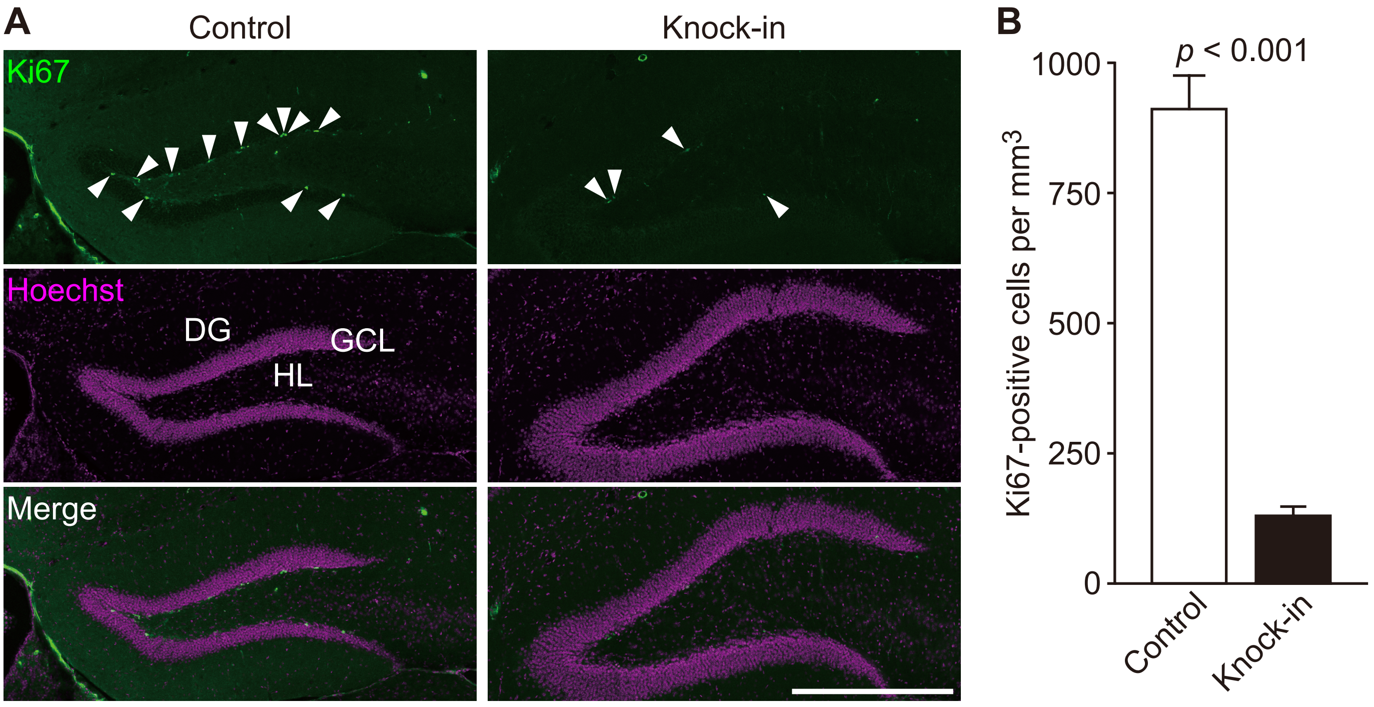

Supplementary Figure S3. Decrease in cell proliferation in SNAP-25 KI mice.
(**A**) Cell proliferation was analyzed using Ki67 immunohistochemical analysis. Ki67 is expressed during all phases of the cell cycle. Arrowheads indicate Ki67-positive cells. Scale bar, 500 μm. GCL, granule cell layer. HL, hilus. (**B**) Quantification of the number of Ki67-positive cells.

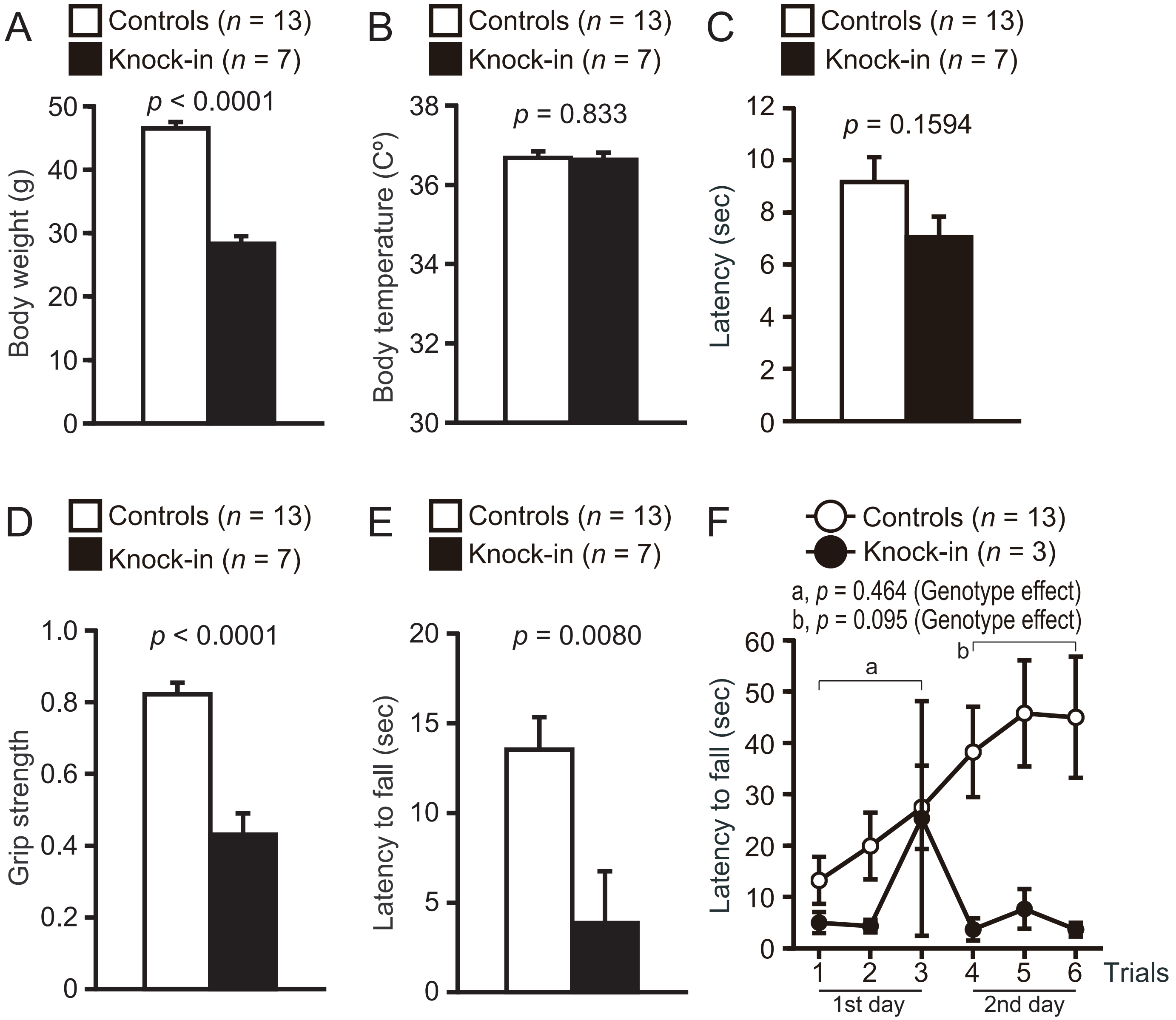

Supplementary Figure S4. Physical characteristics of SNAP-25 KI mice.

Body weight (**A**), grip strength (**D**), and latency to fall in the wire hang test (**E**) of the mutants were significantly decreased compared with the control levels. There were no significant differences in body temperatures (**B**), or the hot plate test (**C**). (**F**) In the rotarod test, although mutants failed to exhibit any significant differences in the latencies to fall both on the first day (Trials 1-3) and the second day (Trials 4-6), mutant mice had a tendency to have shorter latencies to fall on the second day.

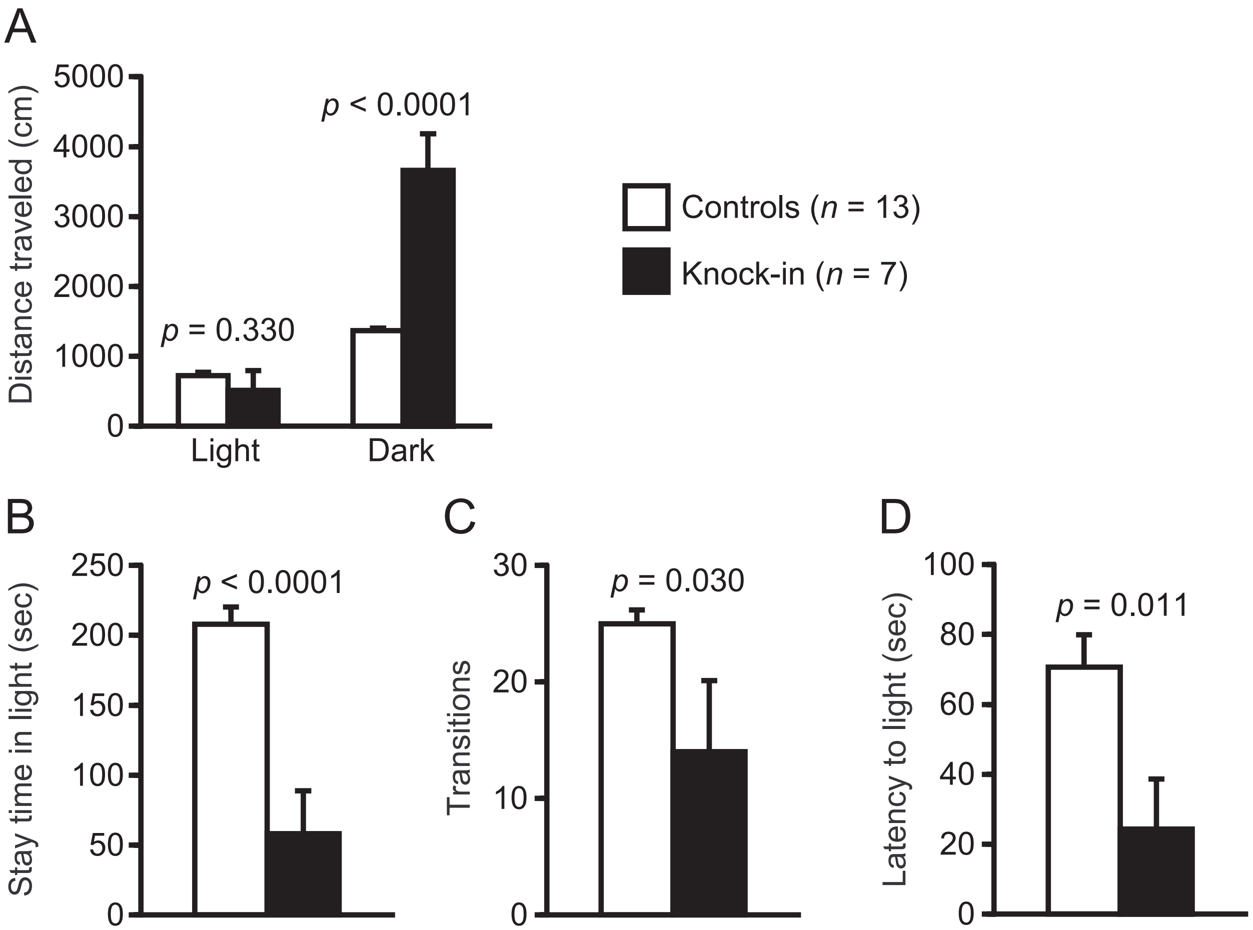

Supplementary Figure S5. Increased anxiety-like behavior in SNAP-25 KI mice during the light/dark transition test. There were significant differences between the genotypes in the distance traveled in the dark box (**A**), time the mice stayed in the light box (**B**), number of transitions between the light and dark boxes (**C**), and latency time before the first entry to the light box (**D**). Note that these results suggest that anxiety-like behavior is increased in SNAP-25 KI mice.

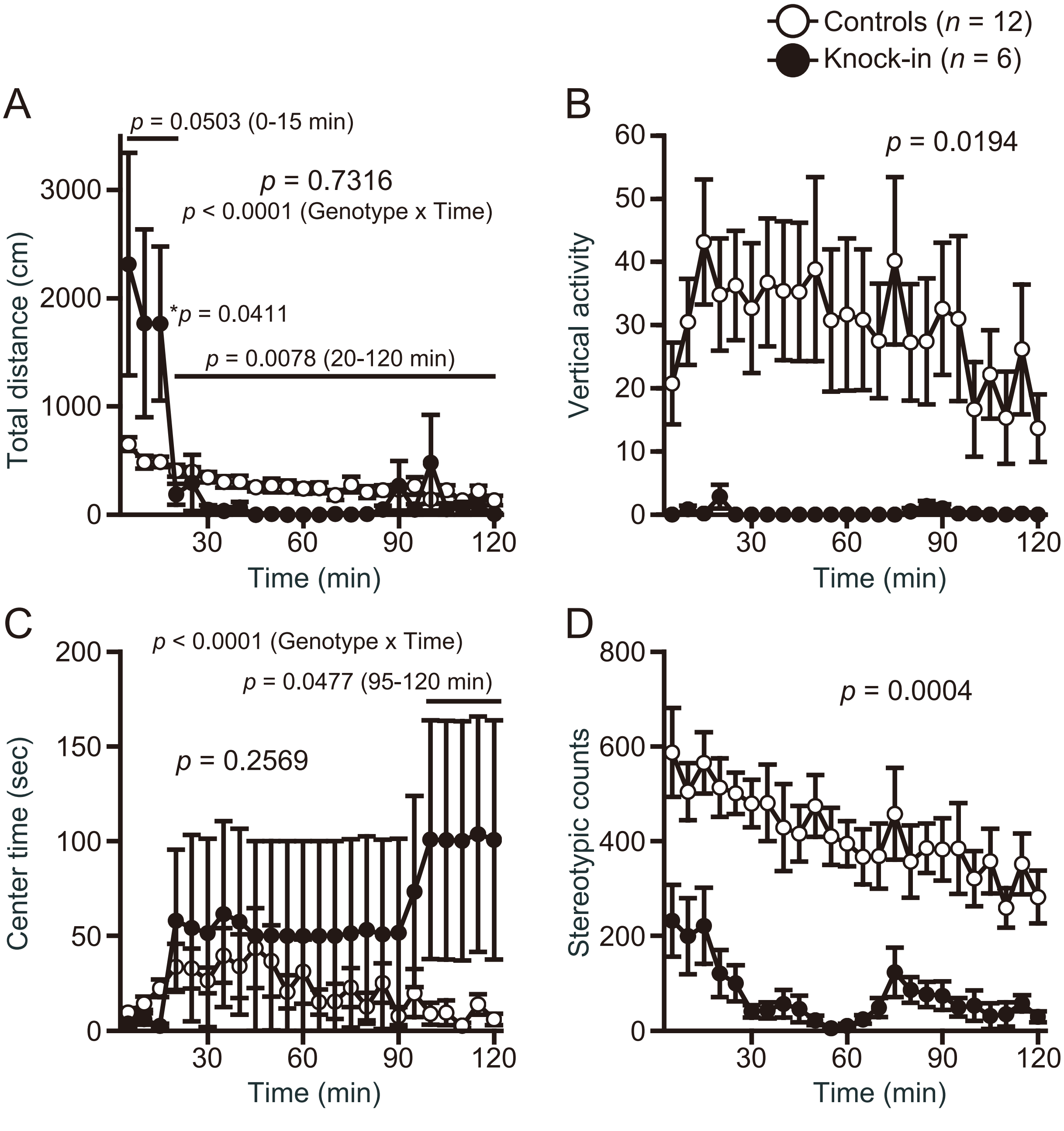

Supplementary Figure S6. Abnormal locomotor activity of SNAP-25 KI mice in an open-field test. (**A**) Total distance traveled over the 120 min period did not change for SNAP-25 KI mice compared with controls. The distance traveled in the first 15 min, however, was generally more for mutants compared with controls, and the distance traveled in the last 100 min was significantly less for the mutants compared with the contols. (**B**) Vertical activity of mutants was significantly decreased. (**C**) SNAP-25 KI mice spent a significantly greater amount of time in the center of the cage during the last 25 min of the test, but there was no significant difference between mutants and controls in the amount of time spent in the center of the cage over the entire 120 min. (**D**) There was a significant decrease in the stereotypic counts for SNAP-25 KI mice.

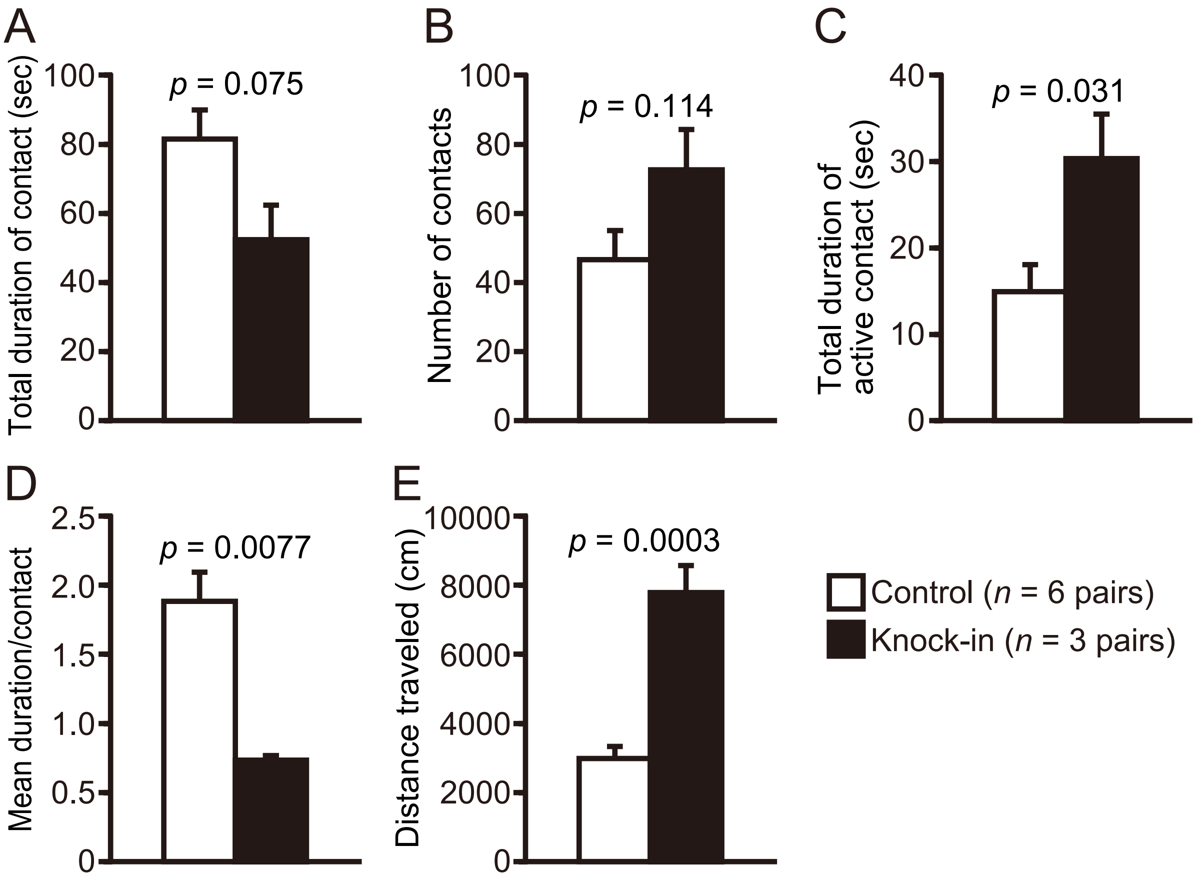

Supplementary Figure S7. Abnormal social behaviors in SNAP-25 KI mice during a social interaction test. (**A**–**E**) A social interaction test in a novel environment (one-chamber social interaction test) was conducted. Total duration of contact (**A**), number of contacts (**B**), total duration of active contact (**C**), mean duration per each contact (**D**), and total distance traveled (**E**) were recorded. During the social interaction test in a novel environment, the total duration of active contact in SNAP-25 KI mice was longer than that of control mice (**C**), but mean duration per contact was decreased in SNAP-25 KI mice (**D**). In addition, SNAP-25 KI mice traveled significantly longer distances (**E**). Total duration of contact tended to be shorter and the number of contacts appeared to be greater for SNAP-25 KI mice. These findings should be the result of hyperactivity, as previous meta-analysis data for over 1000 mice showed that there is a high correlation between locomotor activity and the number of contacts or the total duration of active contact during a social interaction test. SNAP-25 KI mice, *n* = 3 pairs; control mice, *n* = 6 pairs.

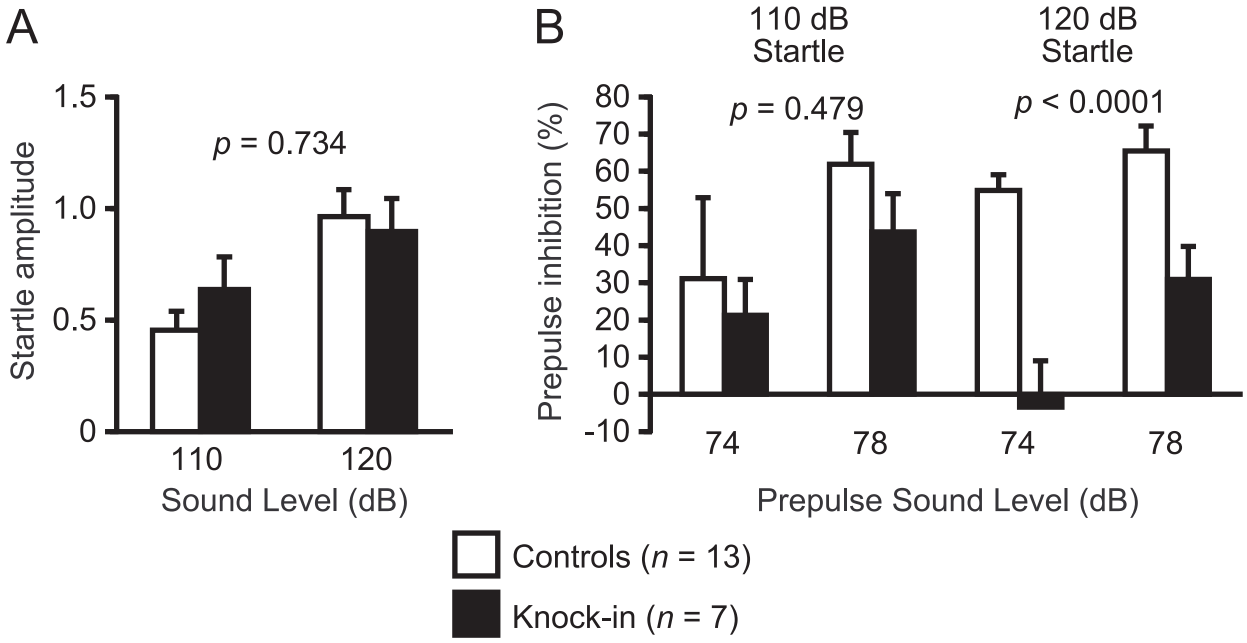

Supplementary Figure S8. Normal startle response and decreased prepulse inhibition in SNAP-25 KI mice. (**A**) No significant differences between control and mutant mice were observed in the acoustic startle response for 110 dB and 120 dB startle stimuli. (**B**) SNAP-25 KI mice showed a significant decrease in prepulse inhibition for 74 and 78 dB prepulse sound levels followed by a 120 dB startle stimulus compared to control mice.

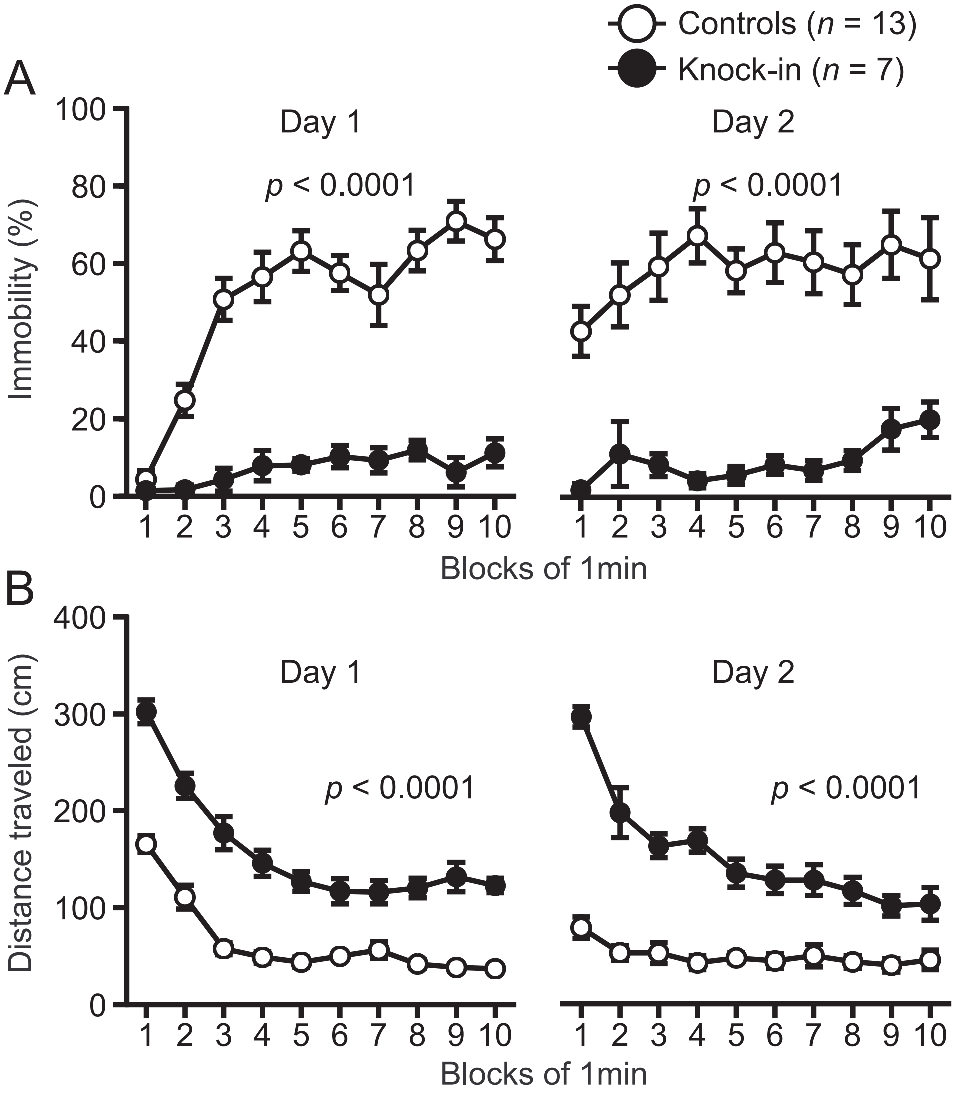

Supplementary Figure S9. Decreased immobility time in SNAP-25 KI mice in the Porsolt forced swim test. (**A**) SNAP-25 KI mice showed significant decreases in immobility time compared to control mice at days 1 and 2. (**B**) SNAP-25 KI mice traveled longer distances than control mice at days 1 and 2.

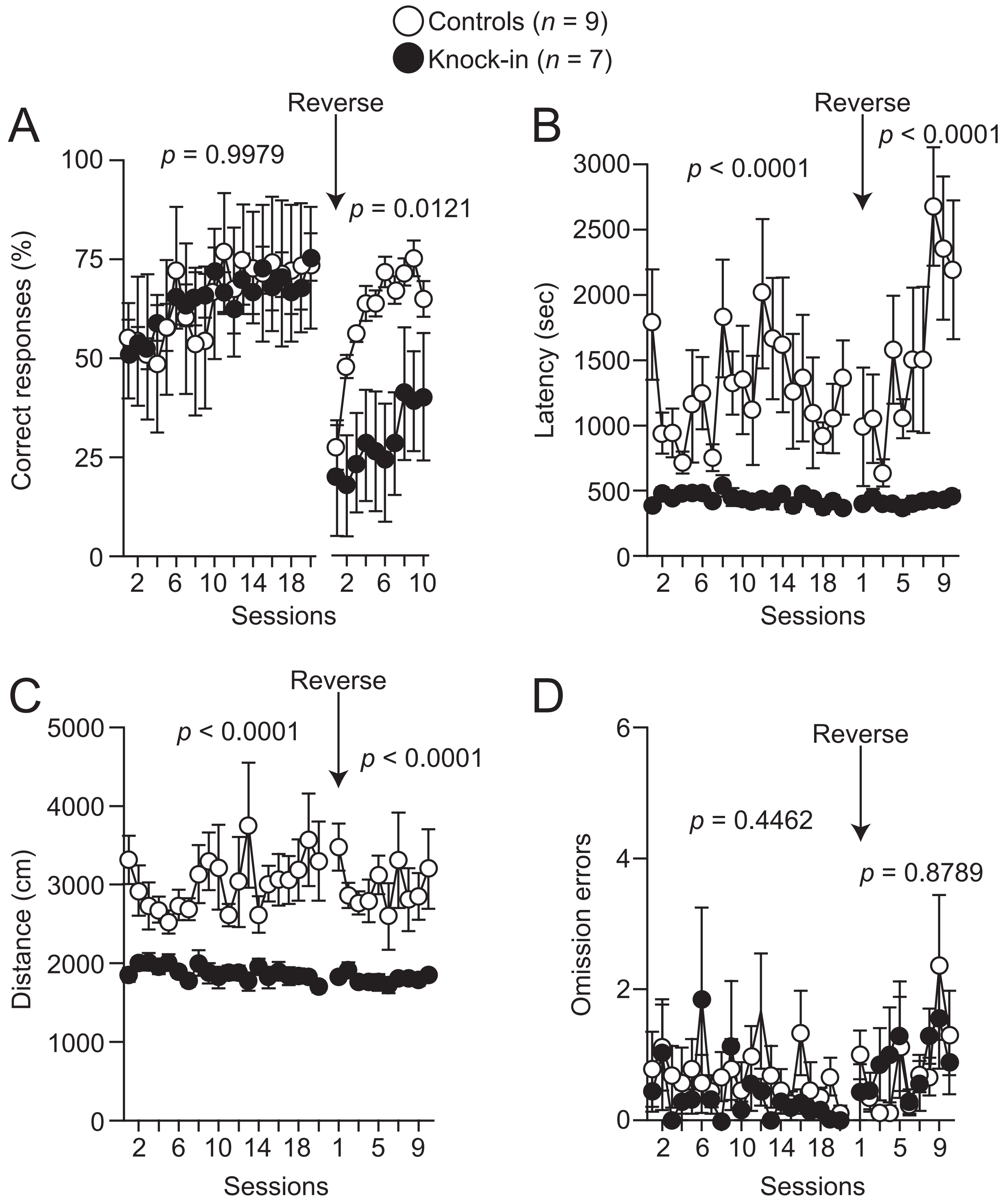

Supplementary Figure S10. T-maze left-right discrimination task. Mice received daily 10 or 20 trials in a session. Data of (**A**) percentage of correct responses, (**B**) latency (sec), (**C**) distance traveled (cm), and (**D**) number of omission errors are represented as means with standard errors for each block of 20 trials, which were analyzed by a two-way repeated measures ANOVA. Mutant mice showed a significantly lower percentage of correct responses than control mice during the reversal learning sessions ($p = 0.0121$).
